# Report of an Amputation in Paris

**Published:** 1857-11

**Authors:** John C. Morfit


### ARTICLE IV.

Dear Sir,—At a time when the subject of *ecrasement* is engrossing so much attention, I have thought it would not be uninteresting to give the following translation of the report of an amputation made by that means in Paris. Heretofore this mode has been made applicable only to tumors, erectile, hœmorrhoidal or fibrous, but Maisonneuve has conceived the idea of applying it to amputation, previously breaking the bone, as in this case, by an ingenious machine which he has invented, and this is the first instance of its use in that way. There is no doubt that this instrument possesses great advantages, not only from avoiding all loss of blood but also from the character of the wound, which scarcely ever suppurates to any great extent, and heals most kindly. One of the great benefits which is claimed from it is the very slight risk of purulent absorption. The case of which I give you a translation was not reported by M. Maisonneuve himself, and is, as you will see, very defective.

Truly yours,

JOHN C. MORFIT.

A man, 33 years old, had been for several years affected with a suppurating white-swelling of the tibis-tarsal articulation, as well as of nearly all the articulations of the tarsus and metatarsus. Various methods of treatment had been tried without success, and the patient put himself under the care of M. Maisonneuve, begging him to amputate the limb. This operation was performed in the following manner:

1st. The patient being put on the operating table, and placed under the influence of chloroform, M. Maisonneuve applying then, upon the inferior third of the leg, a small screw-machine, in the form of a *Serre-nœud*,* broke at a single stroke the two bones on the same level, without contusion of the integuments.

2nd. After this, the operator applied on the flesh about three inches below the level of the fracture, a strong *constrictor*, with which he compressed the tissues moderatly.

3d. While the soft parts were thus strangulated, he incised them with a bistoury about half an inch below the point of constriction, taking care to isolate the bone perfectly.

4th. Seizing then the foot and the lower part of the leg, he makes use of them as a handle to extract the inferior fragment

*The nearest approach to a *Serre-nœud* would be a ligature run through a canula and the end doubled.

of bone which is only held by a few shreds of the soft parts, easily torn.

5th. Finally, he finishes the operation by carrying the constriction as far as the complete division of the skin and all the tissues underneath.

After this operation, which lasted scarcely five minutes, there was not a drop of blood lost. The stump presents a regular appearance, and its extremity is lightly puckered like a purse. Simple dressing was applied, and the patient was carried back to his bed.

*Examination of the piece.*—This examination shows the necessity of the amputation; but what is remarkable, and what our narrator would scarce have believed, although Mr Maisonneuve had, before the operation, declared that we should find such a state of things, is that the tibia and fibula were broken exactly at the same height and without the slightest splinter.—*Gazette de Hopitaux, Sept. 26th.*

